# Glyphosate infiltrates the brain and increases pro-inflammatory cytokine TNFα: implications for neurodegenerative disorders

**DOI:** 10.1186/s12974-022-02544-5

**Published:** 2022-07-28

**Authors:** Joanna K. Winstone, Khyatiben V. Pathak, Wendy Winslow, Ignazio S. Piras, Jennifer White, Ritin Sharma, Matthew J. Huentelman, Patrick Pirrotte, Ramon Velazquez

**Affiliations:** 1 Arizona State University-Banner Neurodegenerative Disease Research Center at the Biodesign Institute, Arizona State University, 797 E Tyler St, Tempe, AZ 85287 USA; 2 School of Life Sciences, Arizona State University, Tempe, AZ USA; 3 Arizona Alzheimer’s Consortium, Phoenix, AZ USA; 4 Integrated Mass Spectrometry Shared Resources (IMS-SR), City of Hope Comprehensive Cancer Center, Duarte, CA USA; 5 Cancer & Cell Biology Division, Translational Genomics Research Institute, Phoenix, AZ USA; 6 Neurogenomics Division, Translational Genomics Research Institute, Phoenix, AZ USA

**Keywords:** Glyphosate, Aminomethylphosphonic acid, TNFα, C57BL/6J, Neuroinflammation

## Abstract

**Background:**

Herbicides are environmental contaminants that have gained much attention due to the potential hazards they pose to human health. Glyphosate, the active ingredient in many commercial herbicides, is the most heavily applied herbicide worldwide. The recent rise in glyphosate application to corn and soy crops correlates positively with increased death rates due to Alzheimer’s disease and other neurodegenerative disorders. Glyphosate has been shown to cross the blood–brain barrier in in vitro models, but has yet to be verified in vivo. Additionally, reports have shown that glyphosate exposure increases pro-inflammatory cytokines in blood plasma, particularly TNFα.

**Methods:**

Here, we examined whether glyphosate infiltrates the brain and elevates TNFα levels in 4-month-old C57BL/6J mice. Mice received either 125, 250, or 500 mg/kg/day of glyphosate, or a vehicle via oral gavage for 14 days. Urine, plasma, and brain samples were collected on the final day of dosing for analysis via UPLC–MS and ELISAs. Primary cortical neurons were derived from amyloidogenic APP/PS1 pups to evaluate in vitro changes in Aβ_40-42_ burden and cytotoxicity. RNA sequencing was performed on C57BL/6J brain samples to determine changes in the transcriptome.

**Results:**

Our analysis revealed that glyphosate infiltrated the brain in a dose-dependent manner and upregulated TNFα in both plasma and brain tissue post-exposure. Notably, glyphosate measures correlated positively with TNFα levels. Glyphosate exposure in APP/PS1 primary cortical neurons increases levels of soluble Aβ_40-42_ and cytotoxicity. RNAseq revealed over 200 differentially expressed genes in a dose-dependent manner and cell-type-specific deconvolution analysis showed enrichment of key biological processes in oligodendrocytes including myelination, axon ensheathment, glial cell development, and oligodendrocyte development.

**Conclusions:**

Collectively, these results show for the first time that glyphosate infiltrates the brain, elevates both the expression of TNFα and soluble Aβ, and disrupts the transcriptome in a dose-dependent manner, suggesting that exposure to this herbicide may have detrimental outcomes regarding the health of the general population.

**Supplementary Information:**

The online version contains supplementary material available at 10.1186/s12974-022-02544-5.

## Background

Herbicides are a ubiquitous part of our environment [1]. Glyphosate (*N*-(phosphonomethyl)glycine), the active ingredient in many commercial herbicides, has been the most heavily applied herbicide worldwide since the year 2000, shortly after the 1996 introduction of glyphosate-tolerant crops [2]. Today, over 113 million kilograms of glyphosate are utilized agriculturally each year across the United States [2]. Glyphosate kills weeds and unwanted plants by inhibiting a key enzyme in the shikimate pathway, enolpyruvylshikimate-3-phosphate synthase (EPSPS), preventing aromatic amino acid biosynthesis vital to plants [3]. While currently deemed safe by the United States Environmental Protection Agency (EPS) and European Food Safety Authority (EFSA), recent research indicates that glyphosate can be toxic to the human body [4, 5], which warrants further investigation. The acute effects of herbicides have been extensively studied, however, the long-term complications of exposure remain largely unknown [6]. Of particular concern is that glyphosate has been shown to cross the blood–brain barrier in vitro, yet has not been studied extensively in the brain [5, 7, 8].

Previous research has shown that subacute (6 weeks) exposure to formulation herbicides (0.05–250 mg/kg glyphosate) can result in inflammation of the peripheral body in adult rats [9]. Specifically, glyphosate exposure resulted in an upregulation of C-reactive protein (CRP) in the liver, and cytokines IL-1β, IL-6, and tumor necrosis factor α (TNFα) in liver and adipose tissue of rats [9]. Others have further confirmed that glyphosate increases peripheral blood levels of TNFα [9–11]. TNFα is an inflammatory cytokine released primarily by macrophages and monocytes throughout the body [12]. Macrophages and monocytes are vital immune cells that can be activated in response to cytokines, bacterial lipopolysaccharide, extracellular matrix proteins, and other chemicals [13]. In the central nervous system (CNS), TNFα is largely produced by microglia (the macrophages of the CNS) [14]. However, astrocytes have also been shown to produce TNFα, which is consistent with their involvement in modulating the neuroimmune response [15].

Aberrant TNFα signaling has been implicated in numerous pathological conditions including cancer, rheumatoid arthritis, psoriasis, multiple sclerosis, as well as immune, inflammatory, and neurodegenerative diseases like Alzheimer’s disease (AD) [16, 17]. In the healthy brain, TNFα expression is low in adulthood [17], while in contrast, adult neurodegenerative diseased brains show very high levels of TNFα [18]. Neuroinflammation plays a central role in AD pathogenesis [19] and TNFα specifically has been strongly implicated in the progression of AD [20]. The TNFα death domain pathway is progressively activated in the AD brain and contributes to cellular degeneration [16]. Interestingly, TNFα inhibition has been shown to reduce generation of monomeric Aβ in a murine model of AD [21] and TNFα inhibitors produce sustained clinical improvement in patients with AD [22].

Previous work has shown that administering either 250 or 500 mg/kg/day of glyphosate to male Swiss mice for 3 months resulted in a decrease in body weight, reduced locomotor activity, and increased anxiety and depression-like behaviors [23]. The dosage used in the aforementioned work is based on the no observable adverse effect limit (NOAEL) for chronic (90 days) exposure in mice established by the EPA [24]. The NOAEL is the maximum dose at which there is no significant toxic effect [25]. It should be noted that this dose is significantly higher than typical daily exposure. A recent review found that the average reported urinary levels in occupationally exposed individuals vary from 0.26 to 73.5 μg/L while individuals with environmental exposure had levels ranging from 0.16 to 7.6 μg/L [26]. Even though human exposure levels are below this reference value, the 500 mg/kg/day still holds value in investigating toxicological effects of the compound [27].

Although the work by Ait Bali et al. found changes in brain-related functions, it did not establish whether glyphosate infiltrated the brain. Additionally, given the relationship between glyphosate exposure and TNFα in the body, and the links between TNFα and neurodegeneration, it is imperative to determine if glyphosate exposure results in detectable levels in the brain. The goal of the present study was to determine if persistent exposure to glyphosate leads to its infiltration in brain tissue and assess its effects on TNFα levels in the brain. We show that glyphosate is detectable in brain tissue in animals dosed with various levels of glyphosate. Furthermore, we determined that various doses of glyphosate drive elevated levels of brain TNFα. RNAseq analysis of hippocampal tissues revealed differentially expressed genes in a dose-dependent manner associated with myelination, axon ensheathment, glial cell development, and oligodendrocyte development. In vitro, we find elevations of soluble Aβ_40-42_ and cell death in glyphosate-exposed primary cortical neurons derived from APP/PS1 (a mouse model of AD) pups. Collectively, these results illustrate that glyphosate exposure infiltrates the brain, and subsequent elevations of TNFα may have implications for neurodegenerative disorders such as AD.

## Materials and methods

### Animals

Non-transgenic (NonTg) C57BL/6J mice were obtained from Jackson laboratories (Stock# 000664) and bred in house. We utilized two cohorts of 24 mice, 48 mice total balanced for sex. All protocols were approved in advance by the Institutional Animal Care and Use Committee of Arizona State University and conform to the National Institutes of Health Guide for the Care and Use of Laboratory Animals. Mice were group housed by sex and dose (3 mice per cage) on a 12-h light/dark cycle at 23 °C and were given food and water ad libitum. Mice were aged to 4 months prior to the start of glyphosate or vehicle dosing.

### Glyphosate and dosing

Chemically pure glyphosate (*N*-(Phosphonomethyl)glycine; C3H8NO5P) was purchased from Sigma-Aldrich (product number P9556) and prepared at 0.107 g/L in 1.89 M sodium hydroxide (NaOH). This calculation was made based on giving a 30 g mouse 140 µL of solution containing 500 mg of glyphosate via oral gavage. The solution was adjusted to a pH of 7 and serially diluted using RO water to achieve the lower concentrations. This solution with no glyphosate served as the vehicle. Mice were randomly assigned to receive one of three dosages starting at 4 months of age: vehicle (control) 125 mg/kg, 250 mg/kg, 500 mg/kg of body weight. Dosages were administered daily via oral gavage for a total of 14 days.

### Blood and urine collection

Blood was collected 4 h after the last dosage via the submandibular vein as previously described [28]. Up to 300 µL of blood was collected into KEDTA + vials. Tubes were inverted 8 times and left at room temperature for 90 min. Tubes were then centrifuged at 4 °C at 2200 rpm for 30 min. Clear plasma fluid was then pulled off the top and stored at − 80 °C for later analysis. Urine was collected on the last 3 days of treatment via manual bladder expression as previously described [29]. Urine was collected directly into 1.7-mL Eppendorf tubes and immediately placed on ice. Tubes were then left at room temperature for 10 min prior to centrifugation at 4 °C at 1500 rpm for 3 min. Supernatant was then transferred to a clean tube and stored at − 80 °C for later analysis.

### Brain tissue processing

Mice were perfused at 4.5 months of age, ~ 4 h after the last gavage treatment, with 1 × PBS. For cohort 1 (*n* = 6 mice/dosage group), brains were extracted and halved along the midline into hemispheres, placed in 1.7-mL Eppendorf tubes, and flash-frozen in isopentane (2-methylbutane). Mice from cohort 2 (*n* = 6 mice/dosage group) were also halved along the midline, and the hippocampus and cortex were dissected out and flash-frozen for protein extraction.

### Brain glyphosate and AMPA measurements

Left brain hemispheres from mice were pulverized using the MultiSample BioPulverizer (BioSpec). The powdered brains were weighed and resuspended in 500 µL of LC–MS grade water. Homogenates corresponding to 5 mg of tissue were aliquoted and spiked with 10 ng/g isotopically labeled internal standards of glyphosate (^13^C_2_^15^N Glyphosate, Sigma-Aldrich, St. Louis, MO) and AMPA (D_2_^13^C^15^N AMPA, Sigma-Aldrich). Samples were boiled at 95 °C for 10 min, cooled to room temperature and sonicated using a cup-horn shaped sonotrode (UTR2000, Hielscher Ultrasound Technology, Teltow, Germany) with 2 cycles of 30 s ON and 30 s OFF at 50% amplitude and 1 cycle of 10 s at 65% amplitude. Homogenates were frozen overnight at − 80 °C. Samples were thawed and acidified with formic acid to the final concentration of 0.1% (*v/v*). Samples were centrifuged at 10,000*g* for 10 min at 6 °C followed by lipid removal using a Sep-Pak C18 solid phase 96 well extraction plate (40 mg sorbent, Waters, Milford-MA). The flow through was subjected to LC–MS/MS analysis. Calibration curves were performed in the analyte free mouse brain matrix over a linear range of 0–50 ng/g of glyphosate and AMPA (coefficient of determination *R*^2^ > 0.99). Multiple Reaction Monitoring (MRM) measurements were performed on a Vanquish Duo UHPLC liquid chromatography system coupled to a Thermo TSQ Altis instrument, as described previously [30]. The limit of detection (LOD) (LOD = *t*(*n* *−* 1, t − α = 0.99) * Ss, where Ss is standard deviation from replicate measurements of a spiked-in standard and *t*(*n*−1, *t* − α = 0.99 represents Student’s *t*-value at 99% confidence with *n* − 1 degrees of freedom) was 0.189 ng/g and 0.122 ng/g for Glyphosate and AMPA, respectively, and limit of quantitation (LOQ) was 0.5 ng/g and 0.4 ng/g for glyphosate and AMPA, respectively (Additional file 1: Fig. S1). The LOQ for the assay was defined as the lowest spiked-in standard with a mean accuracy between 70 and 120% and precision less than 20% RSD [31, 32].

### Glyphosate and AMPA measurements in urine

Urine samples were prepared as previously described [30, 31, 33], with minor modifications. Briefly, mouse urine was diluted 10,000 -fold with water containing 0.1% formic acid. Diluted samples were then spiked with isotopically labeled glyphosate and AMPA standards at 6.25 ng/mL. A standard curve was prepared by spiking a commercially available human urine pool (Lee BioSolutions, Maryland Heights, MO) with unlabeled standards at a linear range of 0 to 20 ng/mL and labeled standards at a constant 6.25 ng/mL. LC–MS/MS measurements were performed as described above. The assay was linear (R^2^ > 0.99) over a range of 0–20 ng/mL for both glyphosate and AMPA. The detection (LOD) and quantitation (LOQ) limits for glyphosate were 0.014 ng/mL and 0.041 ng/mL, respectively, whereas AMPA limits were at 0.013 ng/mL (LOD) and 0.040 ng/mL (LOQ), respectively ([30] 2021; Additional file 2: Fig. S2).

### Protein extraction and ELISAs

Flash-frozen tissue (left hemisphere from cohort 1, left hippocampus and cortex from cohort 2) were homogenized in a T-PER tissue protein extraction reagent, and supplemented with protease (Roche Applied Science, IN, USA) and phosphatase inhibitors (Millipore, MA, USA). The homogenized tissues were centrifuged at 4 °C for 30 min. The supernatant was stored at − 80 °C. Enzyme linked immunosorbent assays (ELISAs) were performed using commercially available Mouse TNF alpha SimpleStep ELISA kits purchased from Abcam (ab208348).

### In vitro experiments

Primary cortical neurons were harvested from newborn APP/PS1 pups (*n* = 3 mice/dosage), plated into 6-well dishes and cultured 12 days using the Primary Neuron Isolation Kit from Pierce (Pierce Cat# 88280). Glyphosate was added to the media of the primary neuron cultures at 40 µg/mL, 20 µg/mL, 10 µg/mL and 0 µg/mL (vehicle only). Samples were tested in triplicate. Twenty-four hours after glyphosate introduction, 0.5 mL of media was collected from the triplicate wells of each treatment and frozen for ELISA analysis of Aβ_40-42_. At this same timepoint, the MTT (MTT 3-(4,5-dimethylthiazol-2-yl)-2,5-diphenyltetrazolium bromide) assay was used to assess cell death as previously described [34]. All absorbance values were normalized to the control group (0 µg/mL vehicle).

### RNA sequencing

RNA was isolated from half-brain samples using the RNeasy Kit (Qiagen). Sequencing libraries were prepared with 250 ng of total RNA using Illumina Stranded Total RNA with Ribo-Zero Plus library preparation (Illumina Inc). PCR-enriched fragments were validated on a 2200 TapeStation (Agilent Technologies) and quantitated via qPCR. The final library was sequenced by 100 bp paired-end sequencing on a HiSeq 2500 (Illumina) at the Collaborative Sequencing Center (Translational Genomics Research Institute, Phoenix, AZ).

Raw reads were aligned to the reference genome GrCm38 using STAR v2.7.5b [35], and summarization of counts at the gene level was conducted by means of featureCounts, as implemented in the R-package Rsubread [36]. Quality controls to assess reads amount and mapping were conducted using MultiQC v1.12 [37]. Then, raw counts were imported into DESeq2 v1.34.0 [38] and transformed by variance stabilizing transformation (VST) to conduct principal component analysis (PCA) for further quality controls. Sex-check was carried out using the counts mapping on X and Y chromosomes using a custom R script [39]. Normalization and differential expression were conducted by means of DESeq2, using a Likelihood Ratio Test (LRT) to assess the relationship between different dosages and gene expression, including sex as a covariate. P-values were adjusted for multiple testing using the False Discovery Rate method (FDR). Genes with adjusted *p*-values < 0.05 were considered as statistically significant differentially expressed genes (DEGs). Pathway analysis was carried out using as input the significant DEGs, and running hypergeometric statistics as implemented in the R-package clusterProfiler [40] referencing Gene Ontology, Kegg, and Reactome databases. Cell-specific gene enrichment was conducted using the markers lists obtained from a single cell RNA-seq study from mouse primary visual cortex [41] using the workflow described in Piras et al. [42]. Enrichment of cell-specific genes was conducted by hypergeometric statistics, as implemented in the R-package bc3net. *p*-values were adjusted for pathway analysis and cell-specific gene enrichment analysis for multiple testing using the FDR method.

### Statistical analyses

Data analysis was conducted using GraphPad Prism version 9.0.2 (GraphPad Software). Statistical outliers were identified using the ROUT method in Prism. One urine glyphosate datapoint was found to be a significant outlier and removed from all subsequent analyses. Factorial one-way ANOVAs were used to analyze dependent variables followed by Bonferroni’s corrected post hoc test, when appropriate. Linear correlations were calculated using the Pearson’s *r* coefficient. Examination of descriptive statistics revealed no violation of any assumptions that required the use of any other statistical test. Significance was set to *p* ≤ 0.05.

## Results

**Fig. 1:**
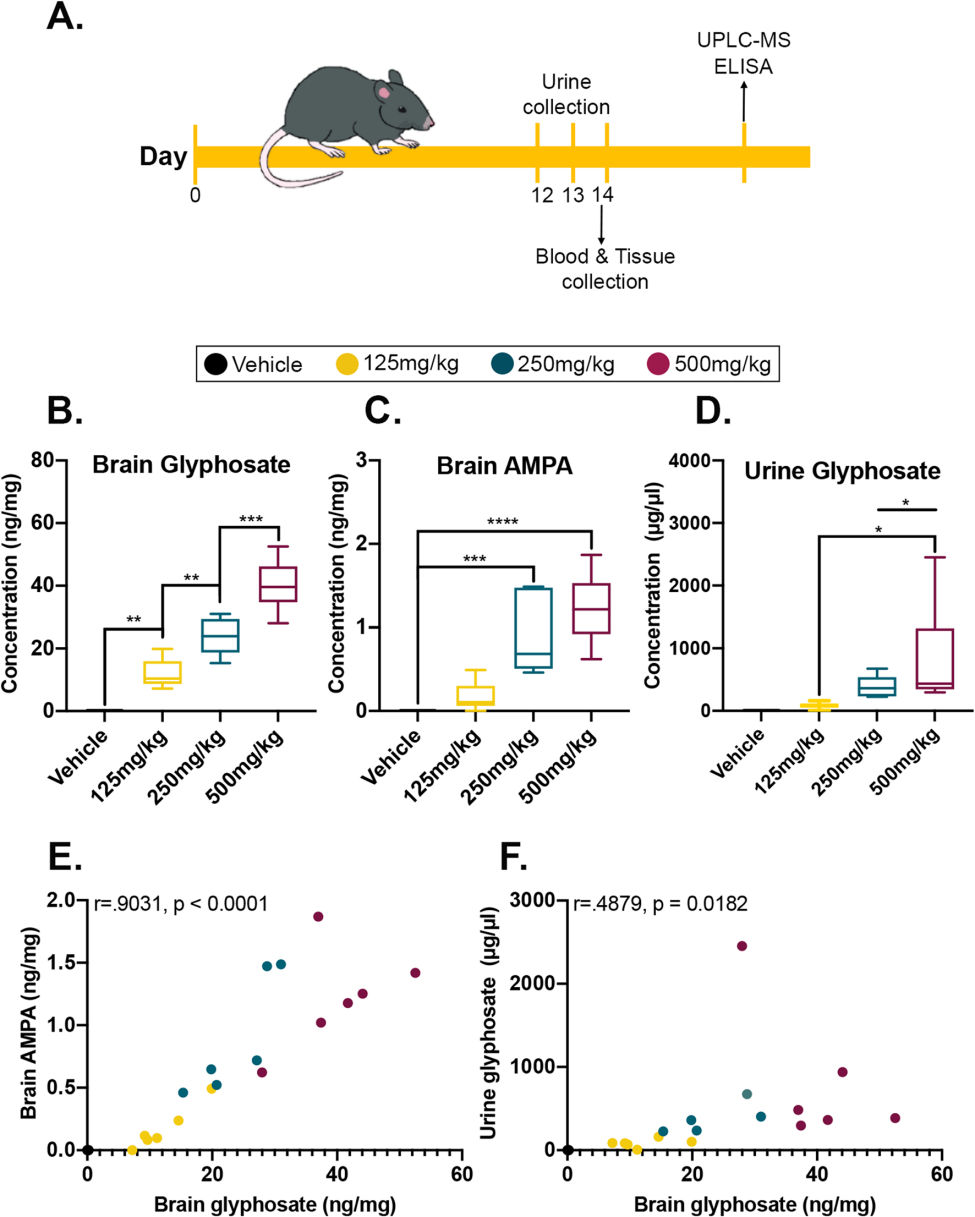
Glyphosate and its major metabolite are detectable in brain tissue. **A** C57BL/6J mice were orally gavaged for 14 days, with urine being collected on the last 3 days. Blood was collected at endpoint, 4 h after the last dosage on day 14, followed by perfusion and postmortem analysis. **B** Levels of glyphosate detected in the brain tissue revealed a significant dose-dependent response between the four groups. **C** Levels of AMPA detected in the brain tissue are elevated in the highest two doses. **D** Level of glyphosate detected in mouse urine is elevated in the 500 mg/kg groups compared to the lower doses. **E** Positive correlation between levels of glyphosate and AMPA in the brain (*p* < 0.0001). **F** Positive correlation between brain and urine glyphosate (*p* = 0.0182). Data in **A**–**C** are presented as boxplots. The center line represents the median value, the limits represent the 25th and 75th percentile, and the whiskers represent the minimum and maximum value of the distribution. *p < .05 **p < .01, ***p < .001, ****p < .0001

To determine whether glyphosate infiltrates the peripheral system and brain in vivo, we delivered either 125, 250, 500 mg/kg/day of glyphosate or a vehicle control for 14 days in two cohorts of 24 mice (*n* = 6 mice/dosage group) via oral gavage (Fig. 1A). Urine was collected from mice at the time of the final dose and four hours later blood samples were collected. Mice were subsequently perfused and had their brains extracted for biochemical assessment.

### Glyphosate is detected in brain tissue and increases in a dose-dependent fashion

To determine whether glyphosate was detectable in perfused brain tissue, we performed UPLC–MS on brain homogenates and urine samples of cohort 1 mice (*n* = 6 mice per group). We found a significant main effect of dosage for brain glyphosate measures (*F*_(3,20)_ = 55.79, *p* < 0.0001; Fig. 1B). Post hoc analysis revealed a significant dose-dependent difference between the four groups, illustrating that increasing dosage resulted in higher glyphosate levels in the brain (*p* < 0.0001). Next, we examined brain levels of glyphosate’s major metabolite, Aminomethylphosphonic acid (AMPA). We found a significant main effect of dosage (*F*_(3,20)_ = 19.18, *p* < 0.0001; Fig. 1C). Post hoc analysis revealed that the 250 mg/kg (*p* = 0.0007) and 500 mg/kg (*p* < 0.0001) dosage groups had higher AMPA expression in the brain compared to vehicle-treated mice. We further observed a significant main effect of dosage for urine glyphosate measures (*F*_(3,19)_ = 4.321, *p* = 0.0175; Fig. 1D). Post hoc analysis revealed that the vehicle (*p* = 0.0198) and 125 mg/kg (*p* = 0.0400) group differed significantly from the 500 mg/kg dosed mice. Lastly, we examined whether brain glyphosate levels correlated with the levels of brain AMPA and urine glyphosate. We found a strong significant positive correlation between brain glyphosate and AMPA (*r* = 0.9031, *p* < 0.001; Fig. 1E), indicating that as brain glyphosate levels increase, so do AMPA levels. We also found a significant positive correlation between urine and brain glyphosate (*r* = 0.4879, *p* = 0.0182; Fig. 1F), illustrating that as urine glyphosate levels increase, so do the levels in the brain. These findings highlight that glyphosate exposure results in detection of both glyphosate and AMPA in the brain, and that the levels of brain glyphosate correlate to the levels detected in urine.

### Glyphosate exposure increases the levels of peripheral blood plasma and brain TNFα in a dose-dependent manner

**Fig. 2:**
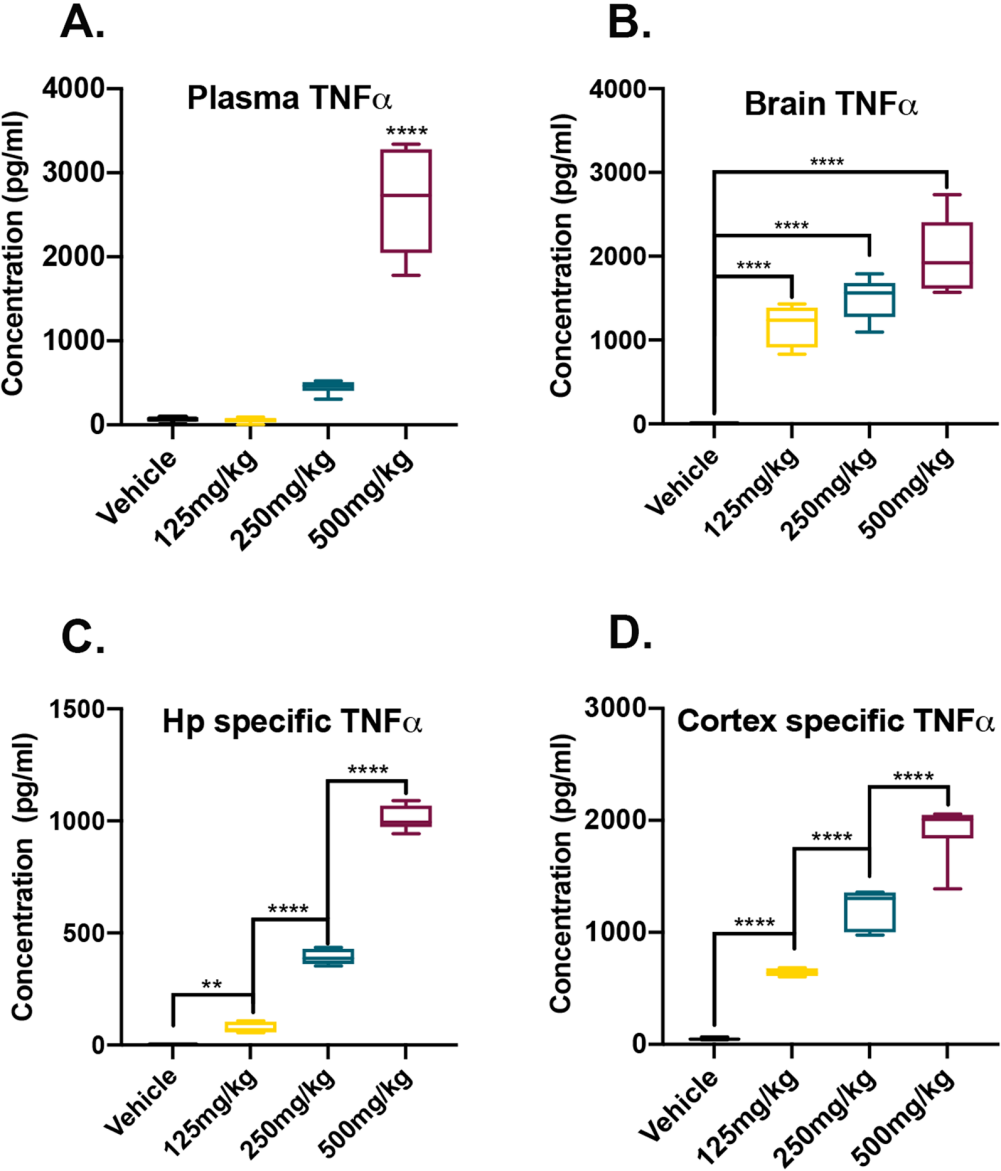
Levels of TNFα are elevated with glyphosate exposure. **A** Plasma concentration of TNFα are significantly elevated after 500 mg/kg daily exposure compared to all other doses. **B** TNFα levels in cohort 1 whole brain homogenates are significantly increased after glyphosate exposure in all three doses. **C** Levels of TNFα are elevated in isolated hippocampal homogenates from cohort 2 mice in a dose-dependent manner. **D** Levels of TNFα in isolated cortical homogenates from cohort 2 mice are elevated in a dose-dependent manner. Data are presented as boxplots. The center line represents the median value, the limits represent the 25th and 75th percentile, and the whiskers represent the minimum and maximum value of the distribution. **p < .01, ****p < .0001

Previous reports have shown that glyphosate exposure increases the levels of peripheral TNFα [9–11]. In the brain, expression of TNFα has been linked to neurotoxicity and cell death [16, 17]. To determine whether glyphosate exposure increases TNFα in the periphery, we analyzed blood plasma from cohort 1 mice via ELISA and found a significant dose main effect (*F*_(3,20)_ = 76.46, *p* < 0.0001; Fig. 2A). Post hoc analysis revealed a significant increase between the vehicle (*p* < 0.0001), 125 mg/kg (*p* < 0.0001) and 250 mg/kg (*p* < 0.0001) groups compared to the 500 mg/kg group. Next, we extracted protein from brain homogenates of cohort 1 mice (*n* = 6 mice/group) and measured the levels of TNFα via ELISA. We found a significant main effect of dosage for TNFα brain homogenate levels (*F*_(3,20)_ = 55.49, *p* < 0.0001; Fig. 2B). Post hoc analysis revealed a significant dose-dependent increase in brain TNFα levels when comparing the vehicle to the 125 mg/kg (*p* < 0.0001), 250 mg/kg (*p* < 0.0001), and 500 mg/kg (*p* < 0.0001) groups. To determine if the increase in brain TNFα is specific to areas affected in neurodegenerative disorders such as Alzheimer’s disease (AD), we dissected out the hippocampus, a structure important for learning and memory [43], and the cortex, from cohort 2 mice (*n* = 6 mice/dosage group). For the hippocampal homogenate analysis, we found a significant main effect of dosage for TNFα levels (*F*_(3,20)_ = 1095, *p* < 0.0001; Fig. 2C). Post hoc analysis revealed a dose-dependent increase in hippocampal TNFα levels when comparing the vehicle to the 125 mg/kg (*p* = 0.0047), 250 mg/kg (*p* < 0.0001), and 500 mg/kg (*p* < 0.0001) groups. Similarly for the cortical homogenates, we found a significant main effect of dosage for TNFα levels (*F*_(3,20)_ = 152.9, *p* < 0.0001; Fig. 2D). Post hoc analysis revealed a dose-dependent increase in cortical TNFα levels when comparing the vehicle to the 125 mg/kg (p < 0.0001), 250 mg/kg (*p* < 0.0001), and 500 mg/kg (*p* < 0.0001) groups. Collectively, these results illustrate that glyphosate exposure increases the levels of pro-inflammatory cytokine TNFα in a brain region-specific manner.

### Brain glyphosate levels correlate with both peripheral blood plasma and brain TNFα levels

**Fig. 3:**
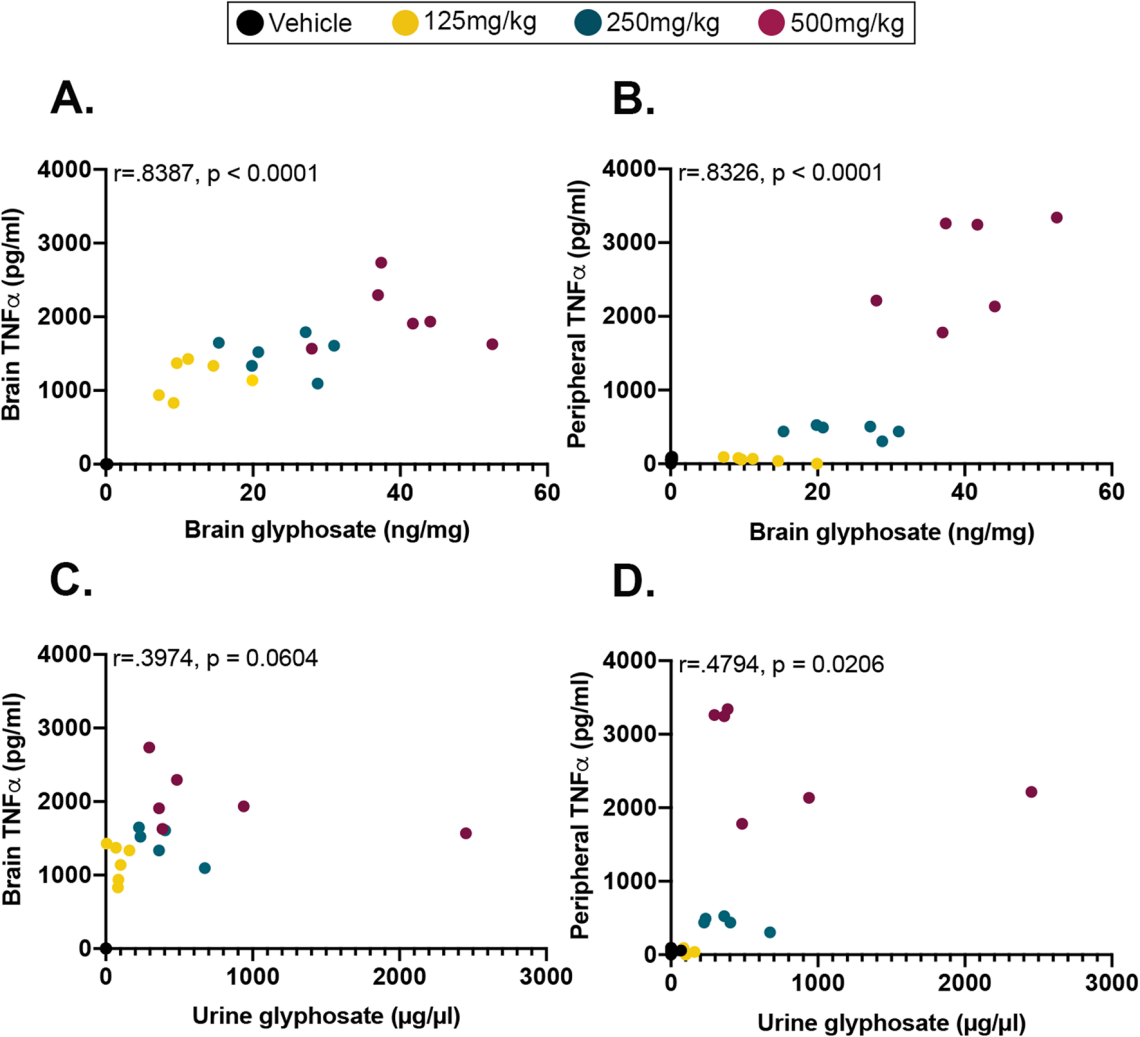
Correlations between urine and brain glyphosate and peripheral blood plasma and brain TNFα measures. **A** Significant positive correlation between brain glyphosate and brain TNFα levels (*p* < 0.0001). **B** Significant positive correlation between brain glyphosate and peripheral blood plasma TNFα levels (*p* < 0.0001). **C** Trending correlation of urine glyphosate and brain TNFα levels (*p* = 0.0604). **D** Significant positive correlation of urine glyphosate and peripheral blood plasma TNFα levels (*p* = 0.0206)

To determine if glyphosate levels detected in brain homogenates and urine correlate with peripheral blood plasma and brain TNFα levels, we performed various linear correlation analyses. We first analyzed brain glyphosate and brain TNFα levels and found a significant positive correlation (*r* = 0.8387, *p* < 0.0001, Fig. 3A), illustrating that as glyphosate goes up, so do the levels of TNFα. We found a significant positive correlation between brain glyphosate and peripheral blood plasma TNFα levels (*r* = 0.8326, *p* < 0.0001; Fig. 3B). Next, we analyzed urine glyphosate and brain TNFα levels and found that it trended toward a significant positive correlation (*r* = 0.3974, *p* = 0.0604; Fig. 3C). Lastly, we analyzed urine glyphosate and peripheral blood plasma TNFα levels, and found a significant positive correlation (*r* = 0.4794, *p* = 0.0206; Fig. 3D), highlighting that as glyphosate increased, so did the levels of TNFα. Collectively, these results show that the levels of urine glyphosate correlate with peripheral TNFα, while brain glyphosate levels correlate with both peripheral blood plasma and brain TNFα levels.

### Glyphosate exposure in APP/PS1-derived primary cortical neurons increases levels of soluble amyloid-β_40-42_ and reduces cell viability

**Fig. 4:**
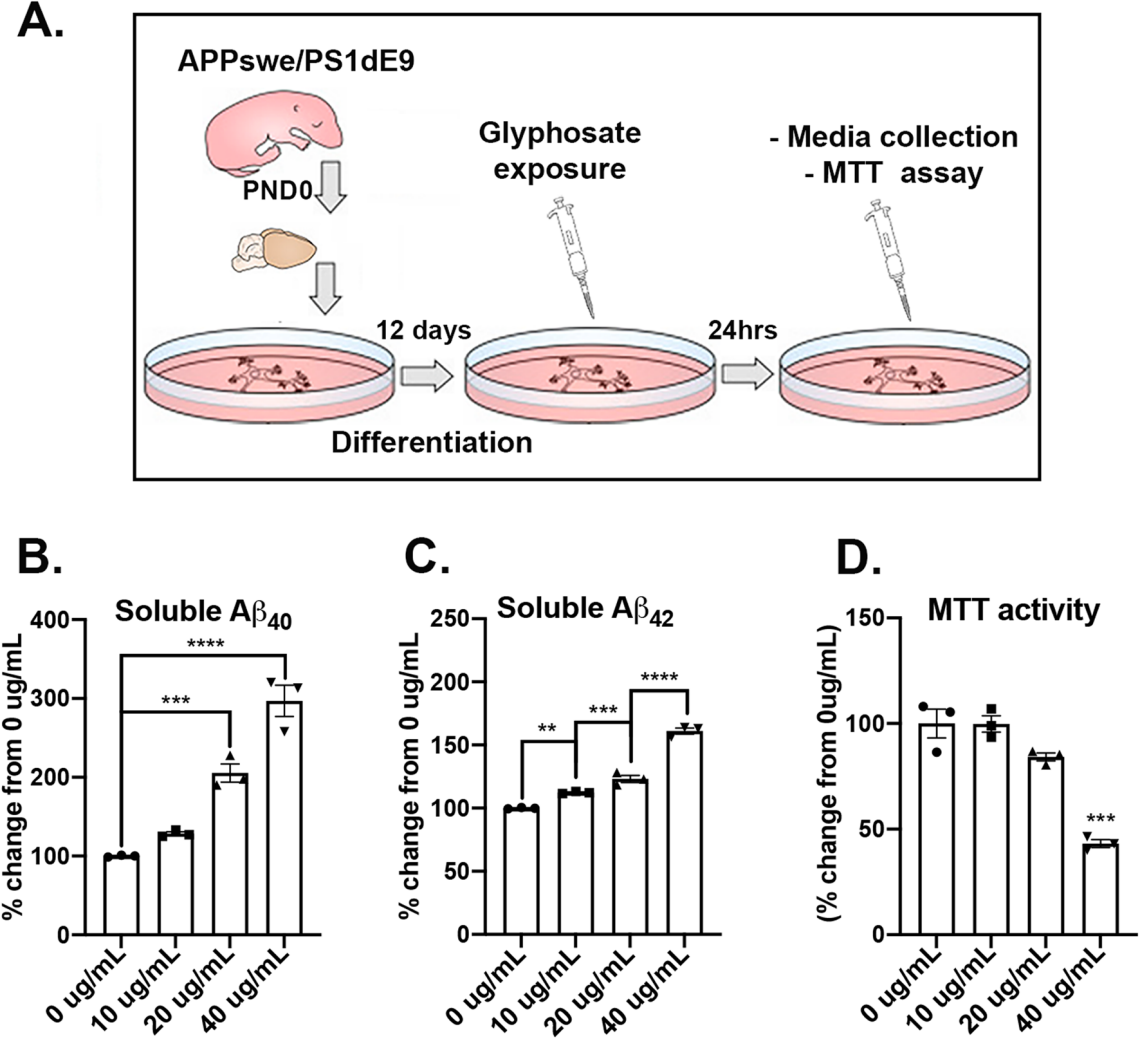
Glyphosate exposure increases Aβ_40-42_ and increase cell death in vitro. **A** Primary cortical neurons were derived from APP/PS1 pups and incubated. Glyphosate was introduced at dosages of 0 µg/mL (vehicle), 10 µg/mL, 20 µg/mL and 40 µg/mL. After 24 h of glyphosate incubation, media was collected, and cell viability was examined. **B** Soluble Aβ_40_ levels are increased in the 40 µg/mL (*p* < 0.0001) and 20 µg/mL (*p* = 0.001) glyphosate treated groups compared to the 0 µg/mL (vehicle) group. **C** Soluble Aβ_42_ levels are increased in a dose-dependent manner in the 40 µg/mL (*p* < 0.0001), 20 µg/mL (*p* = 0.001), and 10 µg/mL (*p* = 0.0092) glyphosate treated groups compared to compared to the 0 µg/mL (vehicle) group. **D** The 40 µg/mL group had a reduced cell viability when compared to the 20 µg/mL (*p* = 0.0007), 10 µg/mL (*p* < 0.0001), and the 0 µg/mL (*p* < 0.0001) group. Scatterplots with bar graphs are means ± SE. *p < .05 **p < .01, ***p < .001

To determine whether glyphosate exposure equivalent to the levels detected in the brain increases the levels of hallmark AD-pathology Aβ and reduces cell viability, we isolated and plated primary cortical neurons from APP/PS1 pups and incubated them with either 0 µg/mL (vehicle), 10 µg/mL, 20 µg/mL or 40 µg/mL of glyphosate (Fig. 4A). At 24 h after exposure, we collected media from culture plates and measured soluble levels of Aβ_40-42_. We found a significant effect of dosage for Aβ_40_ (*F*_(3,8)_ = 59.20, *p* < 0.0001; Fig. 4B). Post hoc analysis revealed that the 20 µg/mL (*p* = 0.001) and 40 µg/mL (*p* < 0.0001) groups showed significant elevated levels of Aβ_40_ than the 0 µg/mL dosage group. Additionally, the 40 µg/mL showed elevated levels compared to the 20 µg/mL (*p* = 0.0029), collectively illustrating a dose-dependent effect. When examining Aβ_42_ levels, we found a significant dosage effect (*F*_(3,8)_ = 202.0, *p* < 0.0001; Fig. 4C). Post hoc analysis revealed that the 40 µg/mL (*p* < 0.0001), 20 µg/mL (*p* = 0.001) and 10 µg/mL (*p* = 0.0092) groups showed elevated levels of Aβ_42_ compared to the 0 µg/mL group. We also found that the 40 µg/mL (*p* < 0.0001) and 20 µg/mL (*p* = 0.0195) groups showed significant elevated levels of Aβ_42_ than the 10 µg/mL dosage group. Like Aβ_40,_ we found that the 40 µg/mL group showed elevated levels of Aβ_42_ compared to the 20 µg/mL group (*p* < 0.0001), collectively illustrating a dose-dependent effect. When we measured cell viability, we found a significant effect of dosage (*F*_(3,8)_ = 41.59, *p* < 0.0001; Fig. 4D). Post hoc analysis revealed that the 40 µg/mL group had a reduced cell viability when compared to the 20 µg/mL (*p* = 0.0007), 10 µg/mL (*p* < 0.0001), and the 0 µg/mL (*p* < 0.0001) group. Collectively, these results show that glyphosate exposure at the levels detected in the brain in vivo are sufficient to increase Aβ_40-42_ levels in a dose-dependent manner and reduce cell viability when tested in vitro using primary cortical neurons derived from APP/PS1 mice.

### Glyphosate exposure results in dose-dependent transcriptomic dysregulation

**Fig. 5:**
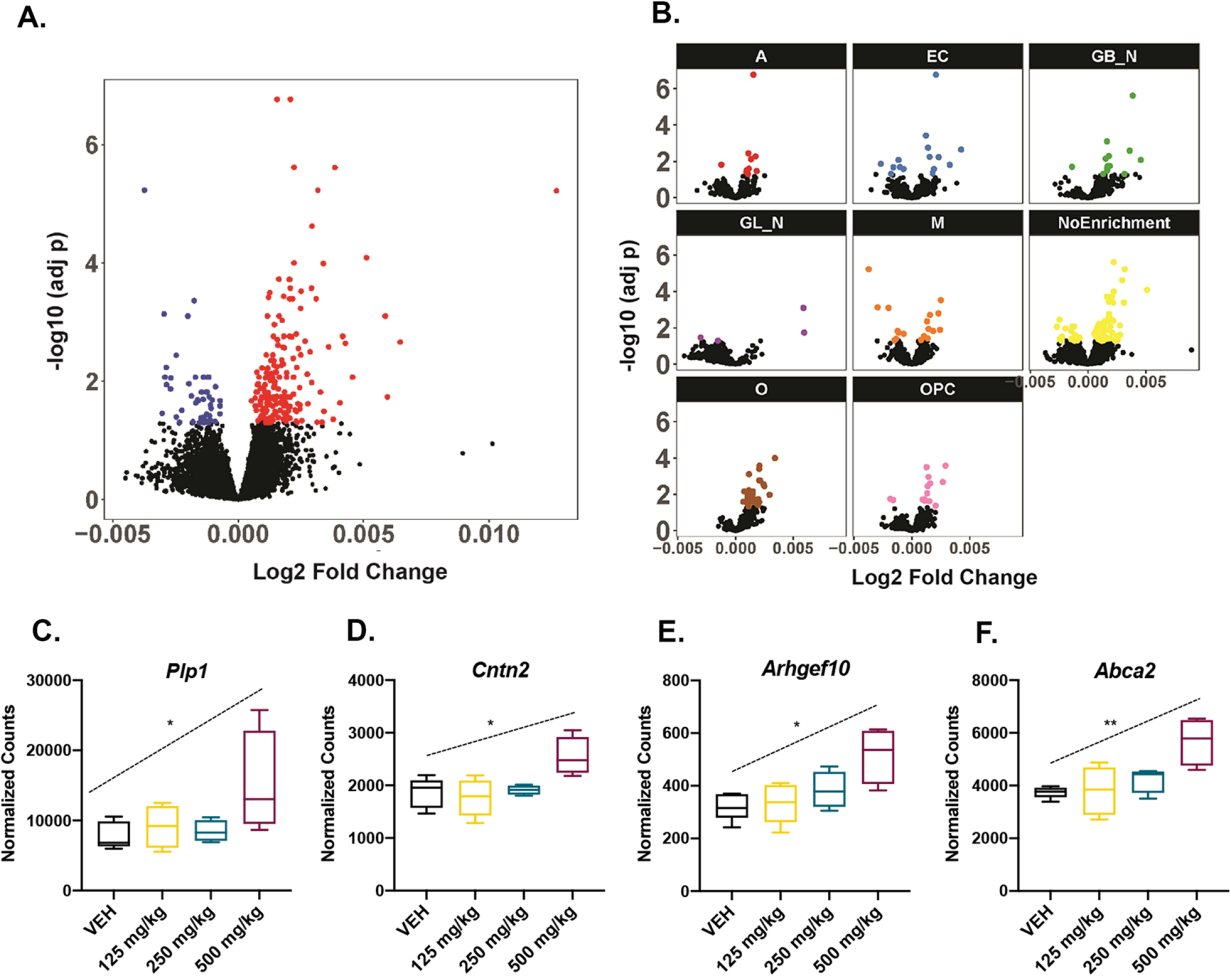
Glyphosate exposure alters the brain transcriptome. **A** Volcano plot showing the differential expression analysis results after dosage regression. Genes in blue and red were downregulated and upregulated, respectively, after glyphosate exposure (adj-*p* < 0.05). **B** Volcano plots showing differential expression in individual cell class following deconvolution analysis. A: astrocytes; EC: endothelial cells; GB_N: GABAergic neurons; GL_N: glutamatergic Neurons; M: microglia; NoEnrichment: unclassified cell type; O: oligodendrocytes; OPC: oligodendrocyte progenitor cells. Colored dots represent dysregulated gene expression. **C** Boxplot of normalized counts for *Plp1* showing significant dose-dependent upregulation (*p* adj. = 0.034). **D** Boxplot of normalized counts for *Cntn2* showing significant dose-dependent upregulation (*p* adj. = 0.029). **E** Boxplot of normalized counts for *Arhgef10* showing significant dose-dependent upregulation (*p* adj. = 0.010). **F** Boxplot of normalized counts for *Abca2* showing significant dose-dependent upregulation (*p* adj. = 0.008)

To characterize changes in the transcriptome following glyphosate exposure, RNA was isolated from brains of mice from cohort 1 and sequenced by 100 bp paired-end sequencing on an Illumina HiSeq 2500. FDR cutoff for differentially expressed genes (DEGs) was set at 5% (< 0.05). Initial dose regression analysis of DEGs showed significant dysregulation of 226 genes in a dose-dependent manner (Fig. 5A; Additional file 3: Fig. S3, Additional file 4: Fig. S4). To examine the functional profile of these genes, pathway analysis was performed using Gene Ontology (GO), Kegg, and Reactome databases. GO and Reactome analysis showed no functional enrichment, and Kegg analysis revealed enrichment of one pathway (mmu05034: Alcoholism) with 11 dysregulated genes (*p*-value = 0.035). Subsequent deconvolution analysis to determine cell-type specific changes in gene expression showed significant enrichment of oligodendrocytes (*p* adj. < 0.000) and GABAergic neurons (*p* adj = 0.006) (Fig. 5B). Pathway analysis of oligodendrocytes and GABAergic neurons was similarly performed, and oligodendrocytes were found to be enriched for four biological processes: central nervous system myelination (GO: 0022010; *p*-adj 0.014), axon ensheathment in the central nervous system (GO: 0032291; *p*-value 0.014), glial cell development (GO: 0021782; *p* adj. 0.033), and oligodendrocyte development (GO: 0014003; *p* adj 0.038). DEGs in these pathways included *Abca2*, *Arhgef10*, *Cntn2*, and *Plp1*, all of which were significantly upregulated with glyphosate exposure in a dose-dependent manner (Fig. 5C–F). Kegg and Reactome showed no significant results for oligodendrocytes, and GABAergic neurons were not enriched in any pathways.

## Discussion

Our results show that glyphosate is detectable in PBS-perfused brain tissue in a dose-dependent manner. This evidence, in conjunction with previous work in isogenic models and postmortem human tissue, suggests that glyphosate can cross the blood–brain barrier [7, 8]. The literature shows neurotoxic effects of glyphosate and its ability to cross blood–brain barrier [8, 23, 44], however glyphosate presence in the brain has not been investigated. In this study, we employed a novel one-step glyphosate extraction method which permitted us to perform LC–MS/MS-based quantification of glyphosate and aminomethylphosphonic acid (AMPA) in brain tissues. Our approach thus provides the first evidence of dose-dependent glyphosate accumulation in the brain.

In addition to the dose-dependent detection of glyphosate in the brain, we detected small amounts of AMPA, the major metabolite of glyphosate, in the brain. This indicates that glyphosate is being degraded in vivo, but further research is required to determine if this is due to metabolism by the gut microbiota, or spontaneous breakdown over time, either of which are plausible [45]. Given that we found glyphosate in the brain, the next step was to determine if glyphosate was inducing inflammatory events within the CNS. TNFα, a marker of inflammation, has been shown to be consistently upregulated in the periphery following glyphosate exposure [9–11]. Our results confirm these reports and show elevated levels of TNFα in the blood plasma of mice exposed to 125, 150, or 500 mg/kg/day of glyphosate. In addition, we found significantly elevated TNFα due to glyphosate exposure in whole brain homogenates. We then isolated the hippocampus and the cortex to probe for TNFα in two regions highly affected by diseases such as AD [46, 47]. We once again found an elevation of TNFα levels in these brain regions. Combined, this data shows that glyphosate can elevate TNFα not only in the peripheral system, but also in key brain regions associated with cognition. Our results illustrate that glyphosate exposure increases the levels of pro-inflammatory cytokine TNFα in the brain, indicating a neuroimmune response to glyphosate exposure.

We also found that glyphosate levels in the brain and urine were positively correlated with peripheral blood plasma and brain TNFα. Specifically, brain glyphosate correlates significantly with both blood plasma and brain TNFα levels. Furthermore, we observed a positive correlation between urine glyphosate and peripheral blood plasma TNFα levels, illustrating that as glyphosate increased, so did the levels of TNFα. As plasma measures of inflammatory response can provide valuable and non-invasive insight into neurological events [48], the correlation between plasma TNFα and CNS measures of both glyphosate and TNFα may have predictive value for neurotoxic levels of glyphosate exposure.

Upon application of comparable glyphosate concentrations observed in brain tissue in vivo to primary cortical neurons in vitro, we found that glyphosate increased cytotoxicity. After 24 h of glyphosate exposure, we found reduced cell viability in the 40 µg/mL dosage group compared to all other dosage groups. This data indicates that the levels of glyphosate detected in the brain in vivo are sufficient to reduce cell viability in a biologically relevant population of cortical neurons lost in AD. This data coincides with the emerging literature showing that an upregulation of pro-inflammatory cytokines can contribute to neuronal damage and loss in neurodegeneration [49]. Not only is glyphosate exposure capable of reducing cell viability, but it also has pathological implications for AD specifically. When we looked at the effects of glyphosate on the production of soluble Aβ_40-42_ in primary cortical neurons derived from APP/PS1 mice_,_ we found that glyphosate elevated soluble Aβ_40_ production at 40 and 20 µg/mL and soluble Aβ_42_ levels at 10, 20 and 40 µg/mL compared to 0 µg/mL. The elevation of Aβ_42_ post-glyphosate exposure is particularly relevant as Aβ_42_ has been shown to be more toxic and fibrillogenic than other forms of Aβ peptide [50]. Collectively, our in vitro experiments show that the levels of glyphosate detected in the brain in vivo after exposure are sufficient to increase cytotoxicity and elevate Aβ_40-42_ levels.

In addition to the elevated Aβ_40-42_ levels, we also show that glyphosate alters gene expression in a dose-dependent manner. Genes dysregulated within oligodendrocytes are functionally associated with key neurological processes including myelination, axon ensheathment, glial cell development, and oligodendrocyte development. Previous studies have shown that oligodendrocytes play a key role in learning and memory, and have been implicated in neurodegenerative disorders that present with cognitive symptoms [51–53]. Interestingly, oligodendrocyte-associated genes, including *Plp1*, have been shown to be dysregulated in human AD postmortem brain samples [54]. *Plp1* dysregulation has also been implicated in other neurodegenerative disorders such as multiple sclerosis [55] and elevation of *Plp1* leads to widespread microglial reactivity and neuroinflammation [56]. The endolysosomal ATP binding cassette transporter *Abca2* has previously been linked with altered neuronal gene expression in AD pathogenesis [57, 58]. In neurons, overexpression of *Abca2* leads to elevated endogenous APP expression and promotes amyloidogenic β-secretase (BACE1) cleavage at the β'-site/Glu11 of Aβ and subsequent γ-secretase cleavage to produce N-terminally truncated Aβ [58]. *Abca2* has been implicated in both early- and late-onset AD [59, 60] and has been suggested as a therapeutic target for AD [58]. In oligodendrocytes however, *Abca2* is thought to be involved in myelination due to its role in sphingolipid metabolism [61–63]. Sphingolipids play an important role in neuron–glia interactions as they regulate formation and stability of myelin [64]. Sphingolipid metabolism has been shown to be deregulated in neurodegenerative disorders including AD [65, 66]. Deregulation of sphingolipid metabolism leads to altered membrane organization and adds to disease pathogenesis [64]. Similarly, Rho guanine nucleotide exchange factor 10 (Arhgef10) is involved in axon ensheathment and myelination [67], while contactin-2 (Cntn2) is a cell-adhesion molecule vital to myelin development [68]. Given that all four of these genes were significantly upregulated in oligodendrocytes following glyphosate exposure, future work will focus on examining the effect of glyphosate exposure on myelin sheath.

Increases in TNFα have been shown to impair oligodendrocyte differentiation, promote mitochondrial dysfunction, and lead to demyelination [69]. As oligodendrocytes are mechanistically important in AD pathology [70], and are impaired by increased levels of TNFα [69], our findings provide insight into a mechanism through which glyphosate may exacerbate neurodegenerative and neuroimmune-related diseases. Specifically, as neuroinflammation has been shown to play a key role in AD initiation and in progression [71], and genome-wide association studies (GWAS) have highlighted several immune genes as risk factors for AD [72, 73]. Glyphosate exposure may lead to an earlier onset or an accelerated progression of AD pathology. Since TNFα is commonly elevated in AD [16], we anticipate glyphosate has an additive effect on pathology and works to exacerbate the neurobiological events underlying this disease. The implications of this potential link would provide causative support to the correlation between glyphosate application to corn and soy crops and the rise in deaths due to AD. While there are many correlations between glyphosate and various illnesses, our goal is to shed light on the correlation between glyphosate application and AD. Future work will focus on uncovering the molecular overlap between glyphosate exposure and AD pathology. Specifically, we will focus on determining if glyphosate exposure is capable of exacerbating amyloid pathology and inducing cell death, in vivo in mouse models of AD.

Although the doses used in this study are above typical daily human exposure [26], our study evaluated the published NOAEL benchmark set forth by the EPA for rodents [24]. These high doses provided valuable information on a potential mechanism of action for glyphosate in AD; however, future work will include more environmentally relevant concentrations of glyphosate. A further limitation of our study is the use of glyphosate as a single agent. Common herbicides provide glyphosate as a formulation with several active ingredients, and some recent studies have focused on glyphosate-based products with complex formulations and revealed associated toxicities [74–77]. Our study centers specifically on glyphosate traversal of the blood–brain barrier and accumulation in the brain, and additional studies are warranted to explore whether complex formulations behave similarly.

## Conclusion

In conclusion, our work demonstrates that glyphosate is capable of infiltrating brain tissue, and that exposure results in increased levels of the pro-inflammatory cytokine TNFα. Additionally, we find that glyphosate dosages similar to those we detected in the mouse brain in vivo are capable of increasing Aβ_40-42_ levels and reducing cell viability in vitro in primary cortical neurons. Brain glyphosate correlates with increased TNFα levels, suggesting that exposure to this herbicide may trigger neuroinflammation in the brain, which may induce changes that are seen in neurodegenerative disorders. This is further supported by our RNAseq findings showing dysregulation of important oligodendrocyte processes known to be affected by elevated levels of TNFα. Collectively, given that a large subset of the population may be exposed to this chemical agent, these results raise awareness of the detrimental effects glyphosate exposure may have on the brain and human health.

### Supplementary Information

**Additional file 1: Figure S1.** Linearity of** A.** glyphosate and ** B.** AMPA over a concentration range of 0-50 ng/g in brain. The area ratio depicts ratio of variable concentrations of glyphosate or AMPA to their respective internal standards (13C215N-Glyphosate or D213C15N-AMPA) with a constant concentration of 10 ng/g.** C**–**F**. Representative MS2 extracted ion chromatograms (EIC) of glyphosate in mice fed at 0 mg/kg, 125 mg/kg, 250 mg/kg, and 500 mg/kg glyphosate.

**Additional file 2: Figure S2. A**, **B.** Linearity of glyphosate and AMPA over a concentration range of 0–20 ng/mL in urine. The X-axis represents glyphosate or AMPA concentrations (0–20 ng) in one mL of urine and Y-axis shows the area ratio of unlabeled standards to their respective labeled standards (13C215N-Glyphosate or D213C15N-AMPA) spiked at a constant concentration of 6.25 ng/mL. **C**–**F.** MS2 scan stage extracted ion chromatograms (EIC) of glyphosate in mice fed at 0 mg/kg, 125 mg/kg, 250 mg/kg, and 500 mg/kg glyphosate.

**Additional file 3: Figure S3.** List of differentially expressed genes following glyphosate exposure.

**Additional file 4: Figure S4.** Heat Map of the top 10% of genes differentially expressed in a dose-dependent manner. Red and blue indicate z-scores with upregulated genes in red and downregulated genes in blue. Dose increases left to right (blue = vehicle control (*n* = 5), Green = 125 mg/kg (*n* = 5), orange = 250 mg/kg (*n* = 4), red = 500 mg/kg (*n* = 4).

## Data Availability

The data that support the findings of this study will be publicly deposited and available.

## Abbreviations

TNFα: Tumor necrosis factor alpha
UPLC–MS: Ultra performance liquid chromatography–mass spectrometry
AD: Alzheimer’s disease
APP: Amyloid precursor protein
ELISA: Enzyme linked immunosorbent assay
PS1: Presenilin 1
Aβ: Amyloid beta
RNAseq: RNA sequencing
EPSPS: Enolpyruvylshikimate-3-phosphate synthase
CRP: C-reactive protein
IL: Interleukin
CNS: Central nervous system
NOAEL: No observable adverse effect level
NonTg: Non-transgenic
NaOH: Sodium hydroxide
RO: Reverse osmosis
KEDTA: Potassium ethylenediaminetetraacetic acid
PBS: Phosphate-buffered saline
LC–MS: Liquid chromatography–mass spectrometry
AMPA: Aminomethylphosphonic acid
MRM: Multiple reaction monitoring
UHPLC: Ultra-high performance liquid chromatography
LOD: Limit of detection
LOQ: Limit of quantitation
RSD: Relative standard deviation
MTT: 3-(4,5-Dimethylthiazol-2-yl)-2,5-diphenyltetrazolium bromide
PCR: Polymerase chain reaction
qPCR: Quantitative polymerase chain reaction
PCA: Principal component analysis
FDR: False discovery rate
LRT: Likelihood ratio test
DEG: Differentially expressed gene
VST: Variance stabilizing transformation
ANOVA: Analysis of variance
GO: Gene Ontology
L–MS/MS: Liquid chromatography with tandem mass spectrometry
PLP1: Proteolipid protein 1
Abca2: ATP binding cassette subfamily A member 2
BACE1: Beta-secretase 1
Argef10: Rho guanine nucleotide exchange factor 10
Cntn2: Contactin-2
EPA: Environmental Protection Agency
