# Correction: Glyphosate infiltrates the brain and increases pro-inflammatory cytokine TNFα: implications for neurodegenerative disorders

**DOI:** 10.1186/s12974-023-02990-9

**Published:** 2024-01-17

**Authors:** Joanna K. Winstone, Khyatiben V. Pathak, Wendy Winslow, Ignazio S. Piras, Jennifer White, Ritin Sharma, Matthew J. Huentelman, Patrick Pirrotte, Ramon Velazquez

**Affiliations:** 1 Arizona State University‑Banner Neurodegenerative Disease Research Center at the Biodesign Institute, Arizona State University, 797 E Tyler St, Tempe, AZ 85287 USA; 2 School of Life Sciences, Arizona State University, Tempe, AZ USA; 3 Arizona Alzheimer’s Consortium, Phoenix, AZ USA; 4 Integrated Mass Spectrometry Shared Resources (IMS-SR), City of Hope Comprehensive Cancer Center, Duarte, CA USA; 5 Cancer & Cell Biology Division, Translational Genomics Research Institute, Phoenix, AZ USA; 6 Neurogenomics Division, Translational Genomics Research Institute, Phoenix, AZ USA

**Correction: Journal of Neuroinflammation (2022) 19:193** 10.1186/s12974-022-02544-5

Following publication of the original article [1], the authors identified an error in the notation of units used in the publication.

All glyphosate and AMPA measurements reported for brain tissue should have been reported as ng/g, instead of ng/mg. This change impacts the axis labels on Fig. 1B, C, E, F, Fig. 3A, B, Supplemental Figure S1A and S1B and two statements in the method section of the publication.

**Fig. 1:**
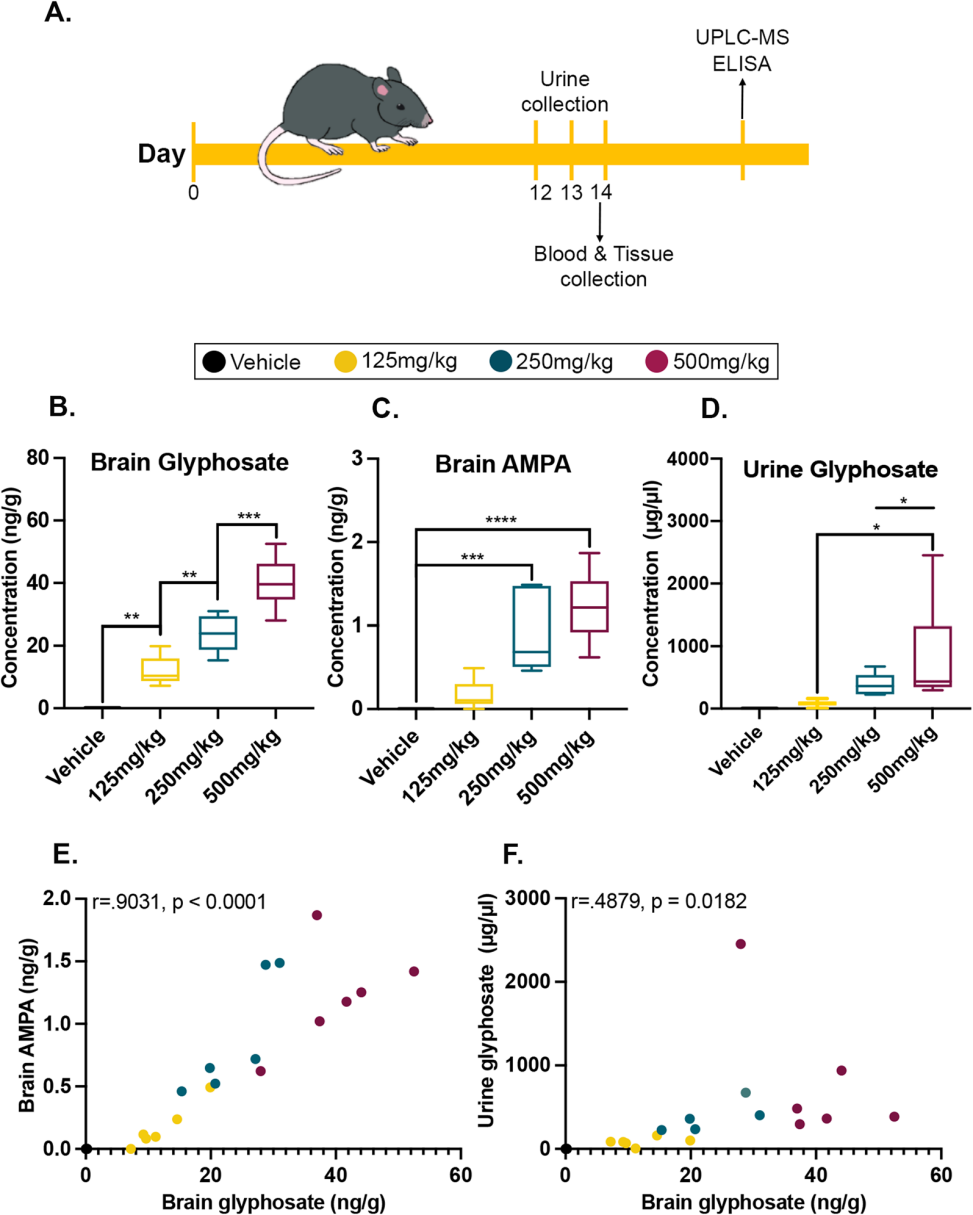
Glyphosate and its major metabolite are detectable in brain tissue. **A** C57BL/6J mice were orally gavaged for 14 days, with urine being collected on the last 3 days. Blood was collected at endpoint, 4 h after the last dosage on day 14, followed by perfusion and postmortem analysis. **B** Levels of glyphosate detected in the brain tissue revealed a significant dose-dependent response between the four groups. **C** Levels of AMPA detected in the brain tissue are elevated in the highest two doses. **D** Level of glyphosate detected in mouse urine is elevated in the 500 mg/kg groups compared to the lower doses. **E** Positive correlation between levels of glyphosate and AMPA in the brain (*p* < 0.0001). **F** Positive correlation between brain and urine glyphosate (*p* = 0.0182). Data in **A**–**C** are presented as boxplots. The center line represents the median value, the limits represent the 25th and 75th percentile, and the whiskers represent the minimum and maximum value of the distribution. *p < 0.05 **p < 0.01, ***p < 0.001, ****p < 0.0001

**Fig. 3:**
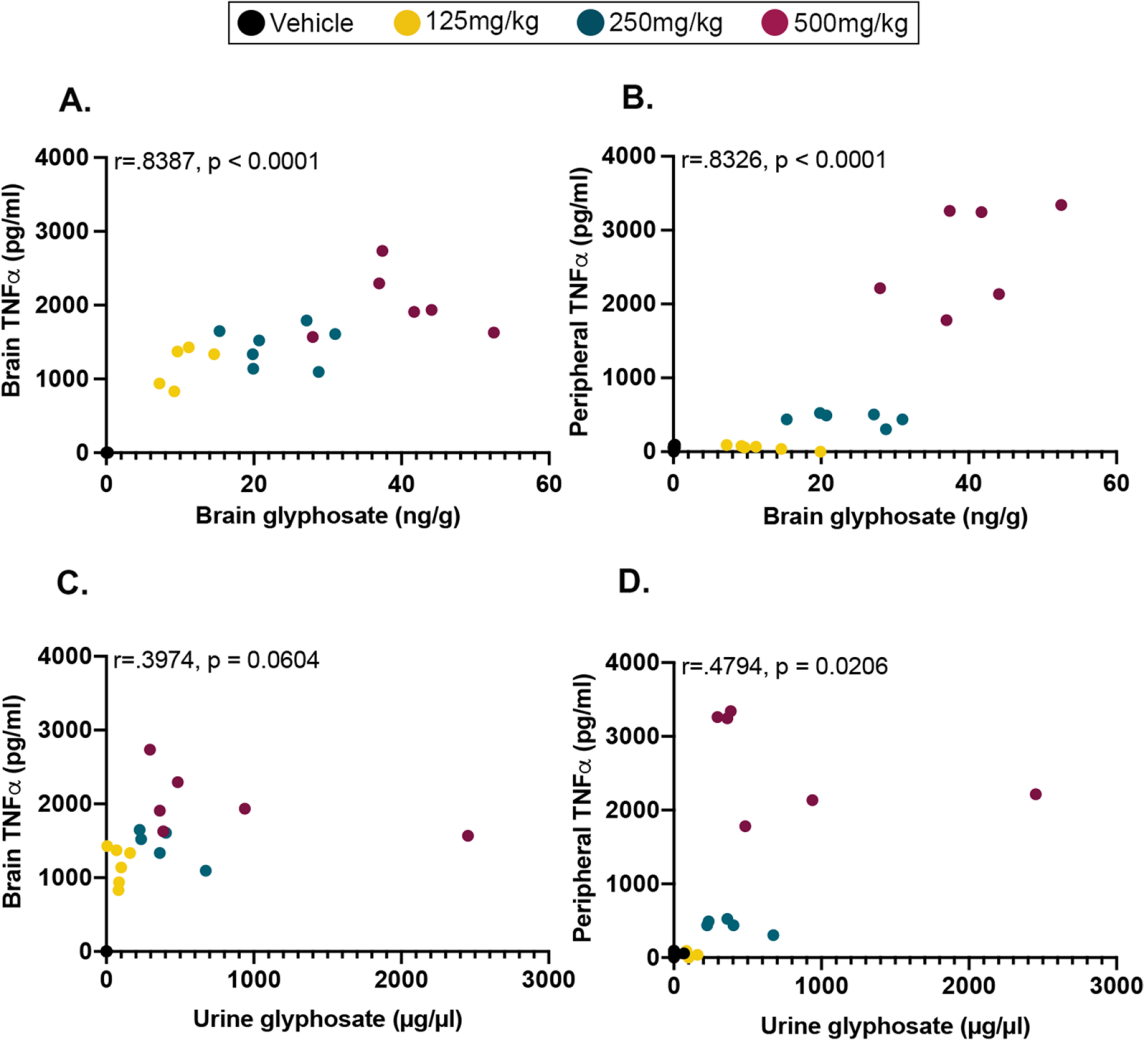
Correlations between urine and brain glyphosate and peripheral blood plasma and brain TNFα measures. **A** Significant positive correlation between brain glyphosate and brain TNFα levels (*p* < 0.0001). **B** Significant positive correlation between brain glyphosate and peripheral blood plasma TNFα levels (*p* < 0.0001). **C** Trending correlation of urine glyphosate and brain TNFα levels (*p* = 0.0604). **D** Significant positive correlation of urine glyphosate and peripheral blood plasma TNFα levels (*p* = 0.0206)

These changes do not impact any findings or conclusions made in this publication. All authors agree to this correction.

Following statements in the methods should be updated as follow:

“Homogenates corresponding to 5 mg of tissue were aliquoted and spiked with 10 ng/g isotopically labeled internal standards of glyphosate (13C215N Glyphosate, Sigma-Aldrich, St. Louis, MO) and AMPA (D213C15N AMPA, Sigma-Aldrich).”

“The limit of detection (LOD) (LOD = t(n − 1, t − α = 0.99) * Ss, where Ss is standard deviation from replicate measurements of a spiked-in standard and t(n−1, t − α = 0.99 represents Student’s t-value at 99% confidence with n − 1 degrees of freedom) was 0.189 ng/g and 0.122 ng/g for Glyphosate and AMPA, respectively, and limit of quantitation (LOQ) was 0.5 ng/g and 0.4 ng/g for glyphosate and AMPA, respectively.”

All figures noted above have been updated in this correction.

The original article has been corrected.

### Supplementary Information

**Additional file 1: Figure S1.** Linearity of **A.** glyphosate and **B.** AMPA over a concentration range of 0–50 ng/g in brain. The area ratio depicts ratio of variable concentrations of glyphosate or AMPA to their respective internal standards (^13^C_2_^15^N-Glyphosate or D_2_^13^C^15^N-AMPA) with a constant concentration of 10 ng/g. **C**–**F**. Representative MS2 extracted ion chromatograms (EIC) of glyphosate in mice fed at 0 mg/kg, 125 mg/kg, 250 mg/kg, and 500 mg/kg glyphosate.
