# Supplementary figures and images for: Glyphosate infiltrates the brain and increases pro-inflammatory cytokine TNFα: implications for neurodegenerative disorders
Source: J Neuroinflammation. 2022 Jul 28;19:193. doi: 10.1186/s12974-022-02544-5 (PMC9331154; doi:10.1186/s12974-022-02544-5)

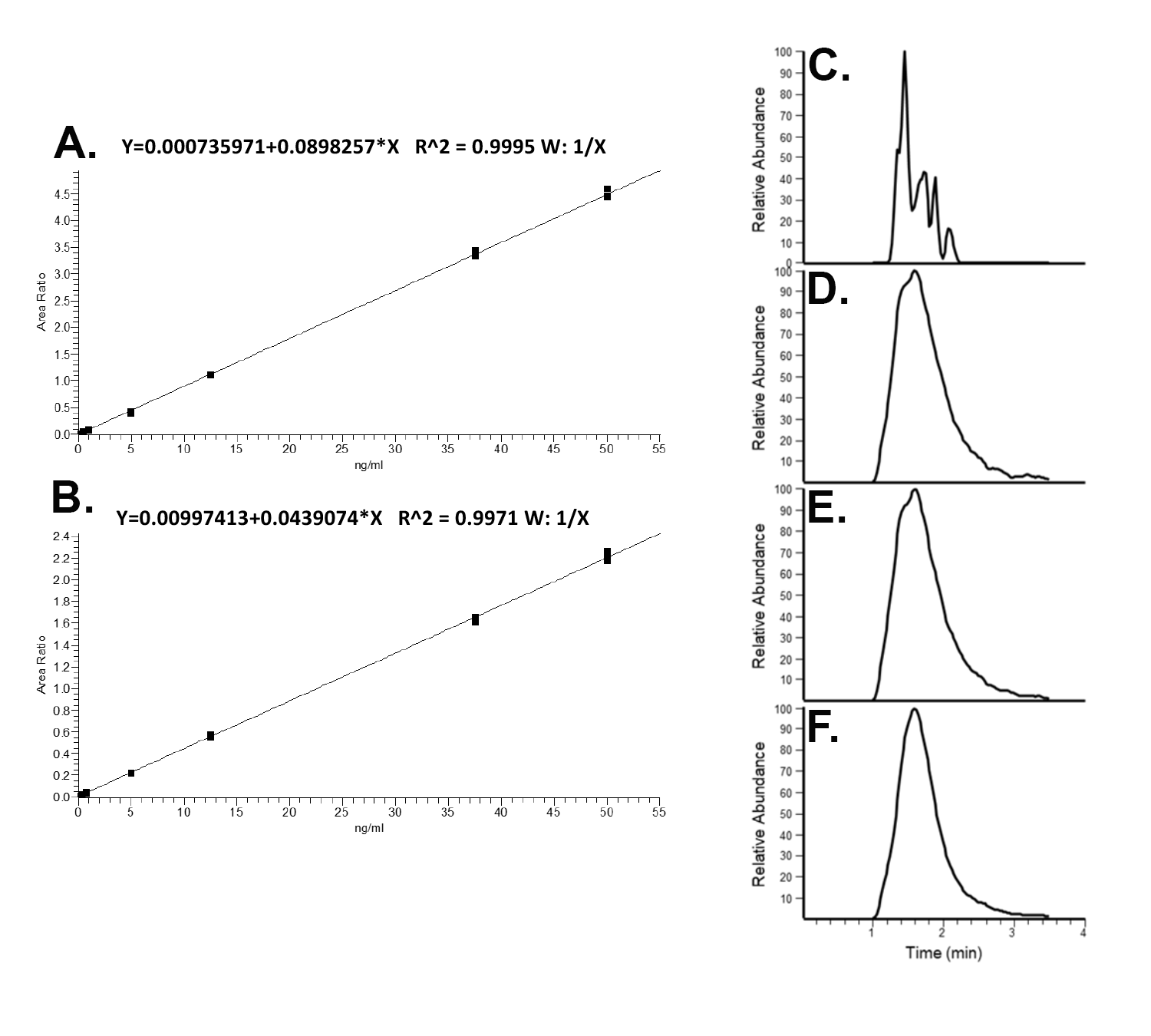

Supplement: Supplementary file 1 — Additional file 1: Figure S1. Linearity of A. glyphosate and B. AMPA over a concentration range of 0-50 ng/g in brain. The area ratio depicts ratio of variable concentrations of glyphosate or AMPA to their respective internal standards (13C215N-Glyphosate or D213C15N-AMPA) with a constant concentration of 10 ng/g. C–F. Representative MS2 extracted ion chromatograms (EIC) of glyphosate in mice fed at 0 mg/kg, 125 mg/kg, 250 mg/kg, and 500 mg/kg glyphosate. [file 12974_2022_2544_MOESM1_ESM.tif]

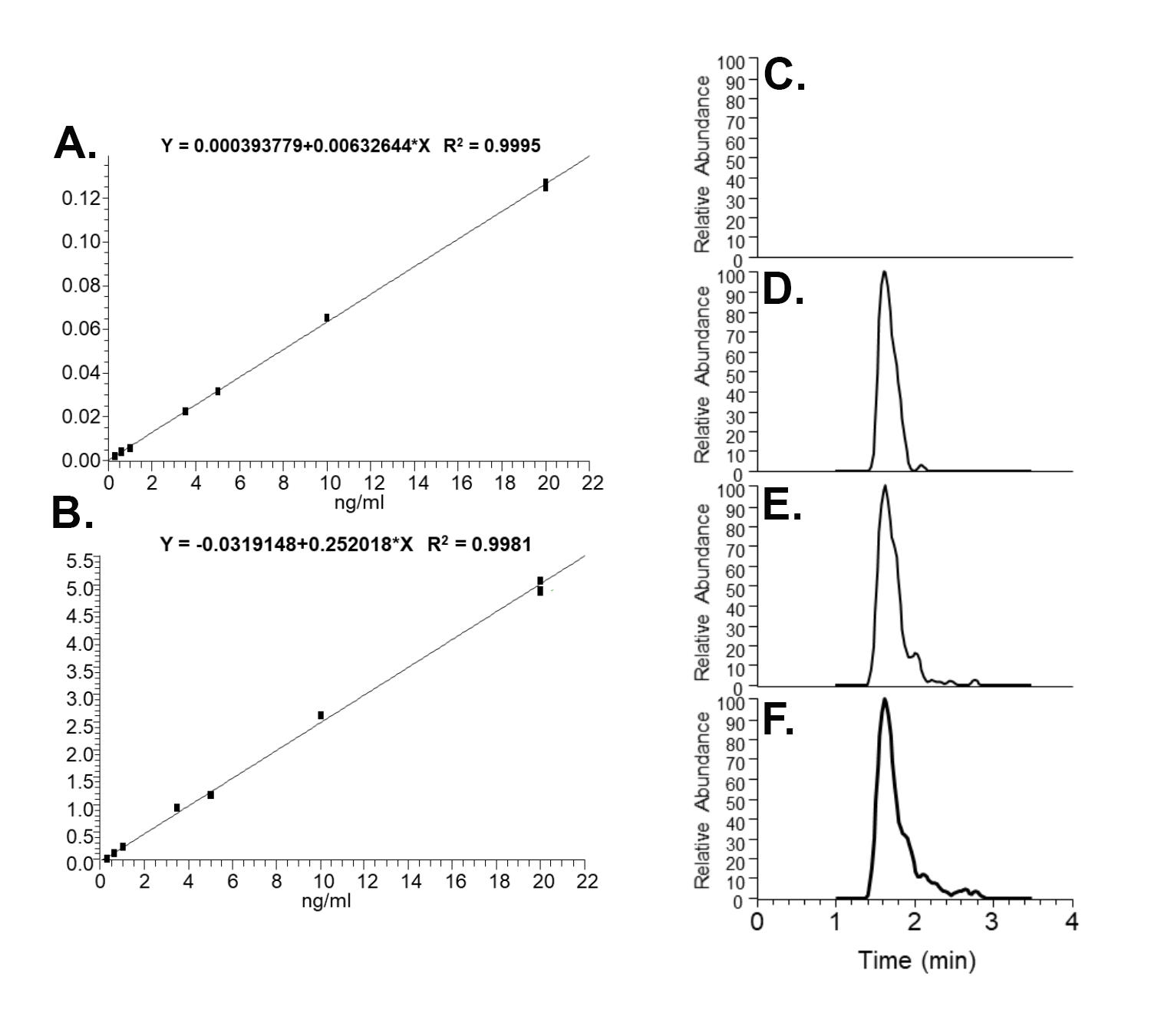

Supplement: Supplementary file 2 — Additional file 2: Figure S2. A, B. Linearity of glyphosate and AMPA over a concentration range of 0–20 ng/mL in urine. The X-axis represents glyphosate or AMPA concentrations (0–20 ng) in one mL of urine and Y-axis shows the area ratio of unlabeled standards to their respective labeled standards (13C215N-Glyphosate or D213C15N-AMPA) spiked at a constant concentration of 6.25 ng/mL. C–F. MS2 scan stage extracted ion chromatograms (EIC) of glyphosate in mice fed at 0 mg/kg, 125 mg/kg, 250 mg/kg, and 500 mg/kg glyphosate. [file 12974_2022_2544_MOESM2_ESM.tif]

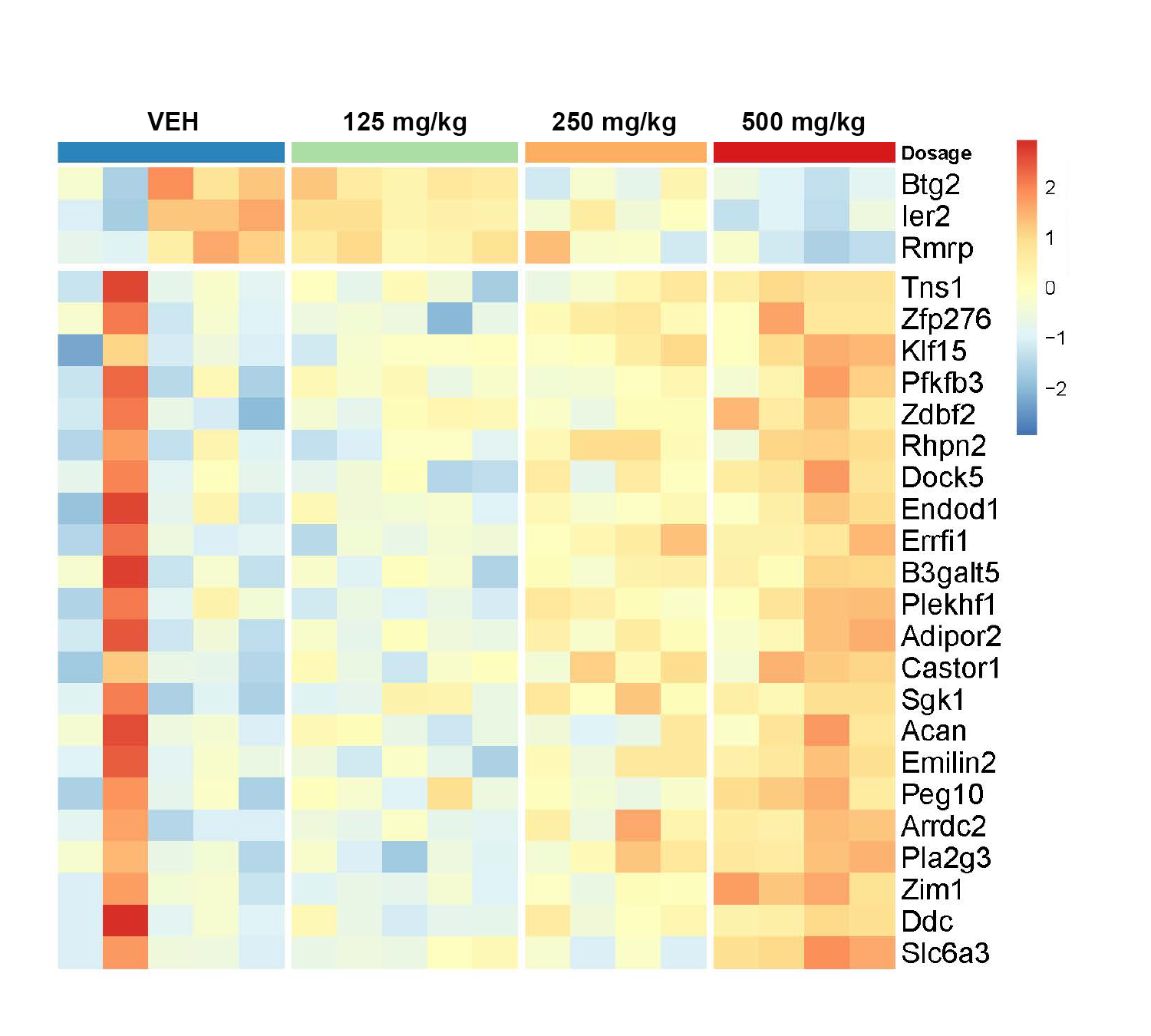

Supplement: Supplementary file 4 — Additional file 4: Figure S4. Heat Map of the top 10% of genes differentially expressed in a dose-dependent manner. Red and blue indicate z-scores with upregulated genes in red and downregulated genes in blue. Dose increases left to right (blue = vehicle control (n = 5), Green = 125 mg/kg (n = 5), orange = 250 mg/kg (n = 4), red = 500 mg/kg (n = 4). [file 12974_2022_2544_MOESM4_ESM.tif]
