# Supplementary material for: Correction: Glyphosate infiltrates the brain and increases pro-inflammatory cytokine TNFα: implications for neurodegenerative disorders
Source: J Neuroinflammation. 2024 Jan 17;21:20. doi: 10.1186/s12974-023-02990-9 (PMC10792902; doi:10.1186/s12974-023-02990-9)
Supplement: Supplementary file 1 — Additional file 1: Figure S1. Linearity of A. glyphosate and B. AMPA over a concentration range of 0–50 ng/g in brain. The area ratio depicts ratio of variable concentrations of glyphosate or AMPA to their respective internal standards (13C215N-Glyphosate or D213C15N-AMPA) with a constant concentration of 10 ng/g. C–F. Representative MS2 extracted ion chromatograms (EIC) of glyphosate in mice fed at 0 mg/kg, 125 mg/kg, 250 mg/kg, and 500 mg/kg glyphosate. [file 12974_2023_2990_MOESM1_ESM.docx]

**Supplemental Figure 1**

**
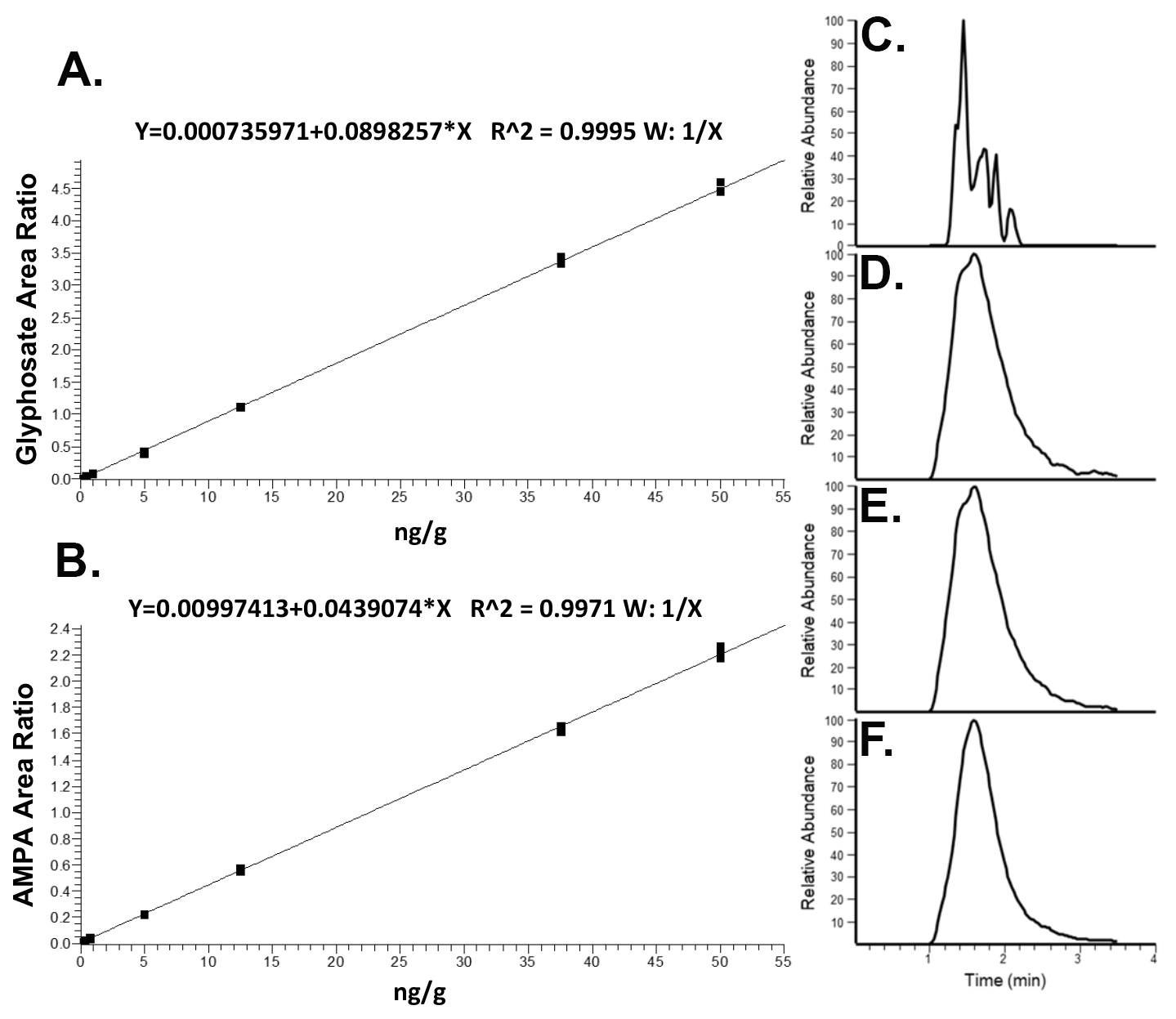
**

**Additional file 1: Figure S1.** Linearity of **A.** glyphosate and **B.** AMPA over a concentration range of 0-50 ng/g in brain. The area ratio depicts ratio of variable concentrations of glyphosate or AMPA to their respective internal standards (^13^C_2_^15^N-Glyphosate or D_2_^13^C^15^N-AMPA) with a constant concentration of 10 ng/g. **C–F.** Representative MS2 extracted ion chromatograms (EIC) of glyphosate in mice fed at 0 mg/kg, 125 mg/kg, 250 mg/kg, and 500 mg/kg glyphosate.
